# Molecular breakpoint cloning and gene expression studies of a novel translocation t(4;15)(q27;q11.2) associated with Prader-Willi syndrome

**DOI:** 10.1186/1471-2350-6-18

**Published:** 2005-05-06

**Authors:** Birgitt Schüle, Mohammed Albalwi, Emma Northrop, David I Francis, Margaret Rowell, Howard R Slater, RJ McKinlay Gardner, Uta Francke

**Affiliations:** 1 Department of Genetics, Stanford University School of Medicine, Stanford CA 94305, USA; 2 Murdoch Children's Research Institute and Paediatrics Department, University of Melbourne, Royal Children's Hospital, Parkville 3052, Victoria, Australia; 3 Department of Child Development and Rehabilitation, Royal Children's Hospital, Parkville 3052, Victoria, Australia; 4 Department of Pathology, King Fahad National Guard Hospital, Riyadh 11426, Saudi Arabia

## Abstract

**Background:**

Prader-Willi syndrome (MIM #176270; PWS) is caused by lack of the paternally-derived copies, or their expression, of multiple genes in a 4 Mb region on chromosome 15q11.2. Known mechanisms include large deletions, maternal uniparental disomy or mutations involving the imprinting center. *De novo *balanced reciprocal translocations in 5 reported individuals had breakpoints clustering in *SNRPN *intron 2 or exon 20/intron 20. To further dissect the PWS phenotype and define the minimal critical region for PWS features, we have studied a 22 year old male with a milder PWS phenotype and a *de novo *translocation t(4;15)(q27;q11.2).

**Methods:**

We used metaphase FISH to narrow the breakpoint region and molecular analyses to map the breakpoints on both chromosomes at the nucleotide level. The expression of genes on chromosome 15 on both sides of the breakpoint was determined by RT-PCR analyses.

**Results:**

Pertinent clinical features include neonatal hypotonia with feeding difficulties, hypogonadism, short stature, late-onset obesity, learning difficulties, abnormal social behavior and marked tolerance to pain, as well as sticky saliva and narcolepsy. Relative macrocephaly and facial features are not typical for PWS. The translocation breakpoints were identified within *SNRPN *intron 17 and intron 10 of a spliced non-coding transcript in band 4q27. LINE and SINE sequences at the exchange points may have contributed to the translocation event. By RT-PCR of lymphoblasts and fibroblasts, we find that upstream SNURF/SNRPN exons and snoRNAs HBII-437 and HBII-13 are expressed, but the downstream snoRNAs PWCR1/HBII-85 and HBII-438A/B snoRNAs are not.

**Conclusion:**

As part of the PWCR1/HBII-85 snoRNA cluster is highly conserved between human and mice, while no copy of HBII-438 has been found in mouse, we conclude that PWCR1/HBII-85 snoRNAs is likely to play a major role in the PWS- phenotype.

## Background

Prader-Willi syndrome (PWS) is a complex neurodevelopmental disorder and a classic example for genomic imprinting in humans. The incidence is about 1 in 10–20,000, and the clinical manifestations include decreased fetal activity, neonatal hypotonia, neonatal feeding difficulties, hyperphagia with obesity, hypogonadism, short stature, small hands and feet, characteristic facial features, and mild to moderate mental retardation. Diagnostic criteria have been proposed [1] and revised recently [2].

**Figure 1:**
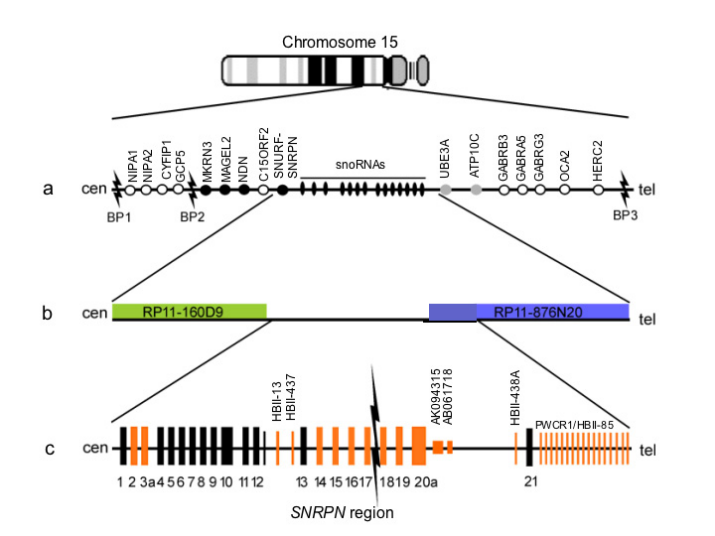
**Mapping the t(4;15) breakpoint and expression patterns of *SNRPN *exons and intronic genes **. **a **. Schematic map of human chromosome region 15q11-q13. Black and gray circles represent imprinted genes, expressed from the paternal or maternal allele, respectively. White circles designate bi-allelically expressed genes. BP1, 2, and 3 indicate the locations of the deletion breakpoint hotspots [43]. **b **. FISH results placed BAC RP11-160D9 highlighted in green (nucleotide position 22577151-22735621) proximal to the translocation breakpoint and RP11-876N20 highlighted in blue (position 22857334-23036552) distal to the breakpoint. Intron 17, comprising nucleotides 22795282 to 22811656, thus is located ~ 63.4 kb downstream of RP11-160D9 and ~ 42 kb upstream of RP11-876N20. **c **. On representation of the *SNRPN *region (not drawn to scale) boxes represent exons and ESTs, lines represent snoRNA copies. Orange boxes and lines indicate exons, ESTs or snoRNAs tested for expression either by RT-PCR or quantitative RT-PCR. Black flash indicates the breakpoint in intron 17 of the *SNRPN *locus.

About 70% of individuals clinically diagnosed with PWS have a ~4 Mb interstitial deletion at 15q11-13 of paternal origin, with clustered breakpoints (BP) at either of two proximal sites (BP1 or BP2) and one on the distal site (BP3) (Fig. 1a). The majority of the remainder have maternal uniparental disomy 15. In about 1 % of the cases, the disease is due to aberrant imprinting and gene silencing. Of these, 14% have small deletions in the imprinting center (IC) region of the paternal allele that abolish the expression of all imprinted paternally-expressed genes in *cis*. In the remainder no demonstrable DNA sequence changes have been observed [3-7]. In Angelman syndrome (AS, MIM#105830), which is usually caused by the same mechanisms affecting the maternal chromosome 15, mutations in the maternal copy of a single gene, *UBE3A *(MIM#601623), encoding a ubiquitin ligase, are detected in about 5% of cases, whereas in PWS, no disease-causing mutations in a single imprinted gene have yet been reported.

Three paternally expressed genes have been identified between BP2 and *SNRPN*. These include *MKRN3/ ZNF127 *(MIM# 603856; Makorin 3 or Zinc finger protein 127) [8,9], *MAGEL2/NDNL1 *(MIM# 605283; MAGE-like 2 or Necdin-like 1) [10,11], and *NDN *(MIM# 602117; Necdin) [12,13] (Figure 1a). The small nuclear ribonucleoprotein polypeptide N (MIM# 182279; SNRPN) gene was the first gene with a known function to be mapped to the PWS/AS deletion region, and is expressed from the paternal chromosome only [14-17]. Multiple alternatively spliced transcripts originate at the *SNPRN *promoter [18-20]. The major SNRPN transcript is bi-cistronic encoding two mRNA species. Exons 1–3 encode a protein product of unknown function called SNURF (SNRPN upstream reading frame). Exons 4–10 encode SmN, a homolog of the SmB/B' protein that binds small nuclear RNAs involved in pre-mRNA splicing. The largest transcripts extend over a ~460 kb genomic region and include a large 3'UTR comprising up to 148 exons [19].

Multiple introns downstream of the SNURF-SNRPN coding region contain C/D box small nucleolar RNA (snoRNA) genes. There are two multi-copy snoRNA clusters (*HBII-52 *and *PWCR1/HBII-85*) [21,22], three single copy snoRNA genes (*HBII-436, HBII-13*, and *HBII-437*), and one snoRNA gene (*HBII-438*) present in two copies that are 240 kb apart [19]. Since the snoRNAs are derived from processed spliced-out introns, their expression is controlled by the SNRPN promoter and is highest in brain. The known function of other C/D box snoRNAs is to guide 2'- O – ribose methylation of ribosomal RNA or small nuclear RNA. This post-transcriptional modification is conserved throughout evolution and is thought to confer increased stability to the small RNA molecules [23]. The modification targets of the imprinted C/D box snoRNAs in the PWS/AS region are still unknown.

Spontaneous chromosome translocations can be extremely valuable for assessing the contributions of individual loci to the phenotype of microdeletion syndromes. Five individuals with features of PWS have been reported who have balanced reciprocal translocations with breakpoints in the PWS/AS deletion region. All of them involve the *SNRPN *locus. The breakpoints are located in intron 2 (proximal, n = 2), disrupting the SNURF/SNRPN coding region, or in exon 20a/intron 20 (distal, n = 3) within the 3'-untranslated region of the long SNRPN transcript. One individual with a proximal and two of three patients with a distal breakpoint meet the diagnostic criteria for PWS (score of 8 or more points) [20,24-28].

Here we report the clinical, cytogenetic and molecular characterization of a 22 year old male with features of PWS who has a different *de novo *balanced reciprocal translocation t(4;15)(q27;q11.2). We mapped the breakpoint to SNRPN intron 17 (position on chr 15: 22803227, UCSC Genome browser May 2004) and determined the expression of snoRNAs on both sides of the breakpoint in cultured fibroblasts and lymphoblasts.

## Methods

### Cytogenetic and FISH analysis

Metaphase spreads obtained from short-term blood lymphocyte cultures and Epstein-Barr virus (EBV)-transformed lymphoblastoid cells (LCL) were processed for high-resolution GTG- banding by standard methods. For FISH studies, Bacterial Artificial Chromosomes (BACs) were sourced from the RPCI-11 library and selected using the UCSC Genome Browser, Assemblies: July 2003 and May 2004). Fluorescence labelling, hybridization procedures and imaging were performed as previously described [29].

### DNA methylation study

Genomic DNA was purified by phenol-chloroform extraction from LCLs from the study subject, a normal control, and a PWS individual (Patient E in [6], Coriell Human Mutant Cell Repository # GM12134). To investigate methylation at exon 1 of SNRPN, 50 μg DNA were used for the bisulfite reaction and PCR with primers according to standard protocols [30,31]. PCR products were separated on a 3% agarose gel, stained with ethidium bromide, and visualized under UV illumination.

### Expression studies by RT-PCR and quantitative RT-PCR

Total RNA was extracted from LCL and primary fibroblast cultures (FB) using RNA Stat 60. The RNA was treated with DNaseI (Roche) and RT-PCR was performed using Superscript II (Invitrogen). Primers were designed for exon-to-exon amplification in an overlapping fashion – where possible – for *SNRPN*, *MKRN3*, *MAGEL2*, *NDN*, snoRNAs, and two ESTs within Intron 20 of *SNRPN *(Table 1).

For a subset of exons in the *SNPRN *gene and the snoRNAs HBII-13, HBII-437, and PWCR1/HBII-85, quantitative RT-PCR assays were performed with SYBR Green I™ dye in an ABI 7700 cycler (Applied Biosystems) by using standard protocols [32,33]. Primers were designed to amplify products of 50 bp in length. GAPDH expression was used as a reference. Each sample was run at least in triplicate. The results were interpreted as described previously [28].

LCL RNA samples from a PWS individual with a microdeletion of the imprinting center (GM12134), a normal individual, an individual with an intrachromosomal triplication of the PWS region on the paternally-derived chromosome 15 (Patient 1 in [34], Coriell Human Mutant Cell Repository # GM12135), and fibroblast RNA from another t(4;15) PWS individual with the breakpoint in intron 2 of *SNRPN *[27,28] served as controls.

### Southern blot analysis

Southern blot analysis was performed according to standard methods with ExpressHyb™ solution (BD Biosciences). Genomic DNA from a normal individual and the t(4;15) carrier was cleaved in a double digestion with restriction enzymes *Nhe*I and *BsaW*I to release a 6.4 kb fragment, and with *Nhe*I and *Apa*I to release a 10 kb fragment in the normal chromosome. The DNA probes were synthesized by PCR from genomic DNA and cloned into a pCRII T/A-vector (Invitrogen). The probes were designed to hybridize within intron 16 (SB-1) and upstream of the *Apa*I restriction site (SB-3) (Table 1).

### Breakpoint cloning with a PCR-based method

Genomic DNA from a normal individual and the t(4;15) carrier was cleaved in a double digestion with restriction enzymes *EcoR*V and *Apa*I, followed by adapter ligation according to the manufacturer's instructions (BD Genome Walker Universal Kit) [35]. A nested PCR reaction with adapter primers and sequence-specific primers was performed and the amplification products were cloned into the pC2.1 T/A-vector (Invitrogen) after gel purification. The clones were sequenced from both directions with universal primers from the vector (M13) and sequence specific primers.

## Results

### Clinical case report

**Figure 2:**
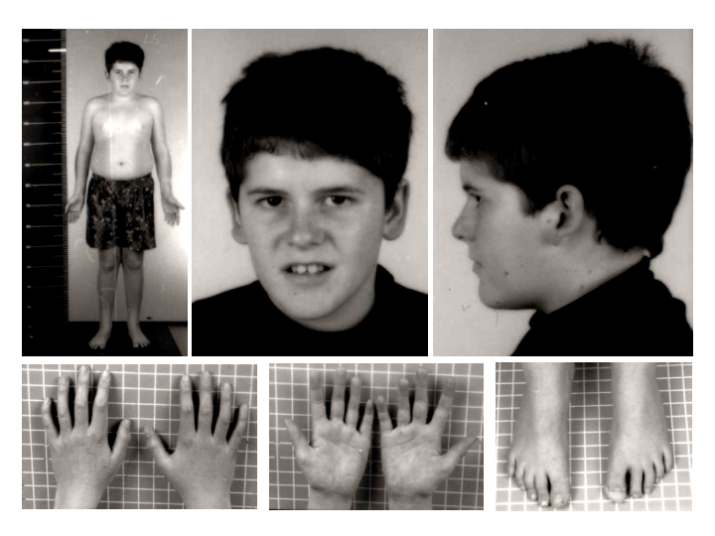
**t(4;15) carrier at 15 years of age **. Note absence of typical PWS facial features and presence of mild truncal obesity.

The patient (Fig. 2) was born at 41 weeks of gestation with a birth weight of 8 lb. Pregnancy was uneventful, but fetal movements were somewhat reduced. In the newborn, poor muscle tone, weak cry, excessive sleepiness, and undescended testes were observed. During infancy, he had poor suck and prolonged feeding times, but his weight gain was satisfactory and he did not require tube feeding. He was suspected to have absence seizures of about 20 seconds duration, along with proneness to giggling, sometimes with eye-rolling. These episodes resolved by four years of age, and an EEG was normal.

He had a left esotropia that was surgically corrected. During childhood, sticky saliva, dry mouth, skin picking and a marked tolerance to pain were noted and have persisted.

Excessive daytime somnolence continued beyond infancy and treatment with amphetamine was started at 9 years of age. A sleep study, at 13 years of age, was normal. In 2002, a further sleep study and a multiple sleep latency tests confirmed the diagnosis of narcolepsy. His daytime sleepiness has continued to respond to dexamphetamine.

Regarding his body weight, there was no rapid weight gain between 1 and 6 years. Around 8 years of age, his interest in food increased, and now he would keep eating if he had unrestricted access to favorite sweet foods. He lives with his parents who help to control his food intake. At 14.5 years, he had small hands and feet, at the 20^th ^percentile and 5^th ^percentile, respectively, and showed mild truncal obesity. His head circumference of 56.7 cm was at the 98^th ^percentile. Brain MRI scan was normal. At age 16 years, his height was 155.7 cm and weight 65 kg. At the age of 22 years, his height is approximately 164 cm and his weight has increased to 90 kg (BMI = 33.5).

At 13 years of age, he was found to have delayed puberty and reduced linear growth velocity with his height falling below the 3^rd ^centile. Treatment with testosterone resulted in improved height gain and genital development. At 15 years of age, he had a left orchidopexy and removal of a dysplastic intra-abdominal right testis. He remains on 6 monthly testosterone implants because of reduced hypothalamic function. He has never been on growth hormone treatment.

Developmentally he had a mild delay in comparison to his older siblings. He attended normal school but had some difficulties due to rigid behaviours and poor peer interactions. Psychological testing (WISC 111, Wide Range Achievement test and BASC self report) revealed an overall normal intellect. However, he had some involuntary fluctuation in attention and significant visual perceptual difficulties, e.g. deficits in visual organization, in making sense of his visual world and transcribing visual material. These perceptual problems have had a significant effect on his learning and social life. At the age of 22, he is attending a mainstream high school requiring extra time and assistance in completing a diploma in information technology. He is good at dismantling computers and installing hardware, and prefers working on his computer to socializing. Hyperphagia and skin picking are still a challenge for him.

### Cytogenetic analysis

**Figure 3:**
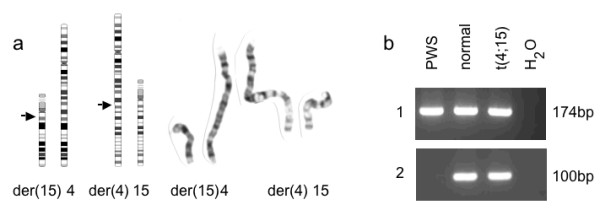
**a. High resolution G-banded ideograms and prometaphase chromosomes of the translocation derivatives and their normal homologs **. An apparently balanced translocation t(4;15)(q27;q11) was identified with arrows indicating band location of breakpoints. **b. DNA methylation analysis of CpG island of SNRPN promoter and exon 1. **1. The 174 bp PCR product is derived from the methylated maternal chromosome. 2. The 100 bp product is derived from the paternal chromosome. PWS: PWS control, Normal: normal control, and t(4;15) carrier; H_2_O: no template control. The t(4;15) carrier shows the normal bi-parental methylation pattern.

High-resolution chromosome analysis showed an apparently balanced reciprocal translocation between the long arm of chromosome 4 and the proximal long arm of chromosome 15. The breakpoints were assigned to chromosome bands 4q27 and 15q11: 46, XY, t(4;15)(q27;q11) (Fig. 3a). Parental chromosomes were normal, indicating that the patient's translocation was *de novo*.

### DNA methylation analysis

To exclude alternative explanations for the phenotype, such as an imprinting defect, DNA methylation analysis was performed. Methylation-specific PCR of the SNURF-SNRPN exon 1 region revealed a normal bi-parental methylation pattern (Fig. 3b).

### Mapping of the translocation breakpoint by FISH

We performed cytogenetic and molecular studies to characterize the breakpoint at 15q11 in detail. Preliminary FISH analysis showed that the breakpoint in 15q11 was located between *D15S11 *and *GABRB3*, which flank the *SNRPN *locus (data not shown). On this basis, a chromosome walking strategy was used across this region to narrow down the breakpoint region. We identified two BACs, RP11-160D9 (current position 22577151-22735621 on UCSC Genome Browser, May 2004 release) and RP11-876N20 (current position 22857334-23036552), that flanked the breakpoint and, thus, mapped it to a ~122 kb interval (Fig. 1b).

### Fine mapping of the breakpoint by SNRPN expression and Southern blot analysis

**Figure 4:**
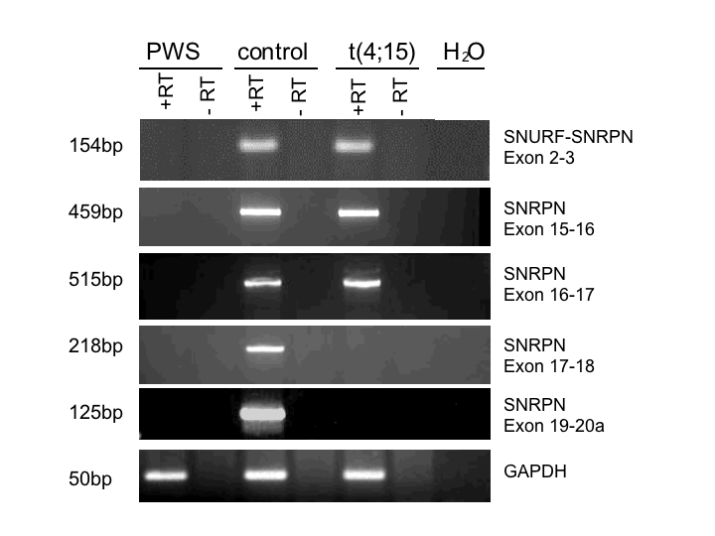
**SNRPN expression analysis by RT-PCR of RNA from LCLs **. On the left, the sizes of the PCR products are shown, and on the right, the location of the primers in SNRPN exons is listed. +RT: with reverse transcriptase; -RT: without reverse transcriptase; H_2_O: no template control. All SNRPN +RT products tested were absent in the PWS control, and present in the normal control. The t(4;15) cells were positive for SNURF/ SNRPN exons 2–3, 15–16 and 16–17 and negative for exons 18 through 20a. GAPDH primers were used as control for the integrity of the cDNA.

**Figure 5:**
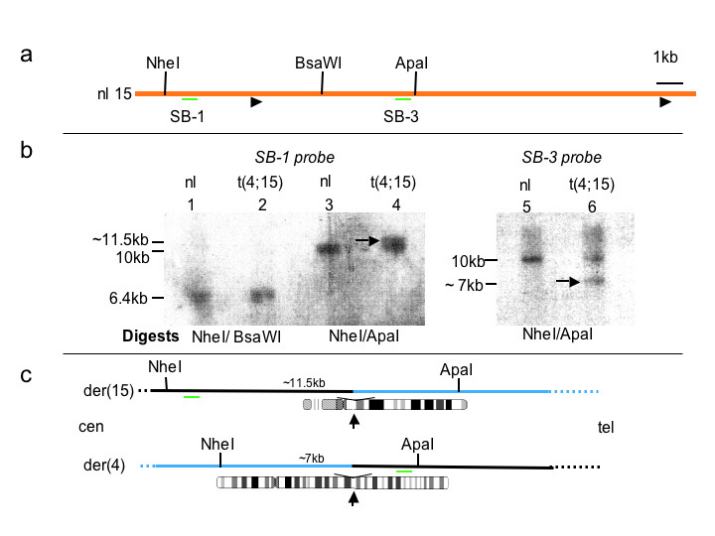
**Southern blot analysis identifies breakpoint in SNRPN intron 17 **. **a **. Restriction map of the intron 17 region of the *SNRPN *gene on the normal chromosome 15. Black arrowheads indicate the boundaries of intron 17. The positions of the two hybridization probes (SB-1 and SB-3) are indicated by green lines. **b **. Lanes 1 and 2 contain double digests with *Nhe*I and *BsaW*I to release a fragment of 6.4 kb, lanes 3 and 4 contain double digests with *Nhe*I and *Apa*I to release a fragment of 10 kb. The membrane was probed with probe SB-1. The arrow indicates an additional band above the 10 kb fragment ~11.5 kb in length. The two bands are not well resolved on the rendition of this blot. This novel fragment is represented in c, upper panel. Lanes 5 and 6 contain double digests with *Nhe*I and *Apa*I to release a 10 kb fragment. The membrane is probed with SB-3. The arrow indicates an additional band of ~ 7 kb. This novel fragment is represented schematically in c, lower panel. **c **. Schematic representation of the junction fragments identified on the Southern blot in b. The upper panel represents the der(15) and the lower panel represents the der(4). Chromosome 15 material is indicated as a black line and material from chromosome 4 as a blue line. Location of restriction sites and of hybridization probes (green lines) are indicated.

To further refine the breakpoint, we carried out quantitative RT-PCR and RT-PCR experiments using RNA from an LCL and skin fibroblasts (FB) for expression of SNRPN transcripts. As shown in Figure 4 and Table 2, we found expression of *SNPRN *exons 2, 3, and 14 to 17, but no expression of exons 18 to 20, and concluded that the breakpoint falls within intron 17. For mapping intron 17, we designed a Southern blot using unique restriction sites. DNA cleaved with *Nhe*I and *BsaW*I showed a 6.4 kb band for the t(4;15) carrier and the normal control (Fig. 5b, lanes 1 and 2), indicating that the breakpoint is located downstream of the *BsaW*I site. Samples doubly digested with *Nhe*I and *Apa*I (Fig. 5b, lanes 3–6), revealed additional bands for the translocation carrier. Besides the expected 10 kb band derived from the normal chromosome 15, there was a ~11.5 kb band in lane 4, detected with the SB-1 probe, and a ~7 kb band in lane 6, detected with probe SB-3 (Fig. 5b). The novel ~11.5 kb band arose from the der(15) chromosome, with an *Nhe*I site on the chromosome 15 portion and an *Apa*I site on the chromosome 4 portion (Fig. 5c, upper panel). The novel band of ~7 kb arose from the der(4) chromosome, with an *Apa*I site on the chromosome 15-part and an *Nhe*I site on the chromosome 4-part. Taken together, these results delimit the breakpoint region to ~3.6 kb between the *BsaW*I and *Apa*I sites (Fig. 5a).

### Breakpoint mapping at the nucleotide level

**Figure 6:**
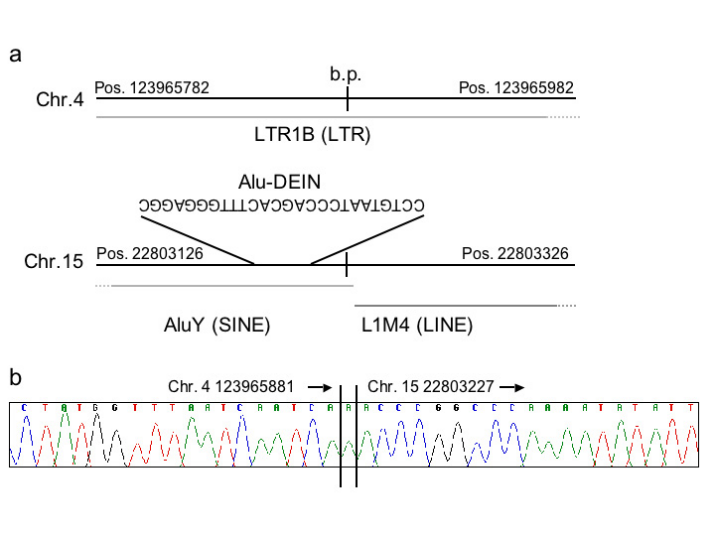
**Repeat sequences surrounding the breakpoint **. **a **. One hundred nucleotides on either side of the breakpoints on chromosome 4 and 15 contain repetitive sequences (grey lines). The Alu-DEIN sequence is located 13–39 bp upstream of the breakpoint on chromosome 15. **b **. Sequence across the breakpoint on the der(4) chromosome reveals an additional A inserted at the breakpoint. Arrows indicate the direction centromere to telomere.

By DNA sequencing, we mapped the breakpoint to *SNRPN *intron 17 (position chr 15: 22803227) and to chromosome 4 at position chr. 4:123965881 (UCSC Genome Browser, May 2004) (Fig. 6). On chromosome 4, a long terminal repeat (LTR) retrotransposon, LTR1B, spans the breakpoint. On chromosome 15, we found a short interspersed element (SINE), AluY, and a long interspersed element (LINE), L1M4, surrounding the breakpoint (Fig. 6a). Thirty-nine bp upstream of the breakpoint on chromosome 15 starts a common 26 bp core sequence of Alu elements (Alu-DEIN) in an inverted orientation. This sequence is known to be involved in gene rearrangements [36]. While the sequence across the breakpoint is contiguous on the der(15), an extra A is inserted on the der(4) chromosome (Fig. 6b). Furthermore, the breakpoint on chromosome 4 falls in a large intron between exons 10 and 11 of a spliced transcript (BC045668). By RT-PCR, we found that this transcript is expressed in fibroblasts, but not in LCLs (data not shown).

### Expression of upstream genes *MKRN3*, *MAGEL2*, and *NDN*

Expression of the three imprinted genes *MKRN3*, *MAGEL2*, and *NDN *upstream of SNRPN was tested by RT-PCR in t(4;15) fibroblasts and found to be indistinguishable from expression in normal control fibroblasts (data not shown).

### Expression of C/D box snoRNAs and intron-encoded ESTs

**Figure 7:**
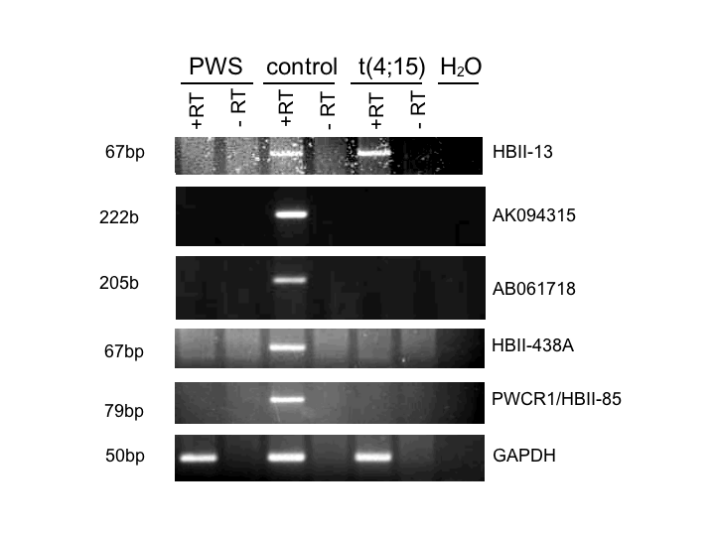
**Expression analysis in LCLs of snoRNAs and two ESTs in intron 20 **. RT-PCR analysis of the C/D box snoRNAs reveals expression of HBII-13, but not of HBII-438A/B, PWCR1/HBII-85 and the two ESTs in intron 20 in the t(4;15) translocation carrier. +RT: with reverse transcriptase; -RT: without reverse transcriptase; H_2_O: no template control.

When testing for the intron-encoded C/D box snoRNAs, we were able to document expression of HBII-13 and HBII-437 and lack of expression for HBII-438A/B and HBII-85/PWCR1 (Fig. 7). By use of a more sensitive method, quantitative real-time RT-PCR, we obtained similar results for the *SNRPN *exons and snoRNAs tested (Table 2). Two ESTs, AK094315 and AB061718 (= HBT8) located in the 30 kb *SNRPN *intron 20 were not expressed in the PWS control [6] and t(4;15) LCLs, but were expressed in the normal control LCL (Fig. 7).

## Discussion

### Breakpoint mapping and mechanism of the translocation event

Dissecting the PWS deletion region and identifying individual genes as responsible for parts of the phenotype represent a challenge because all reported smaller deletions inactivate all imprinted genes on the paternally- derived chromosome 15. Rare reciprocal translocations, therefore, provide unique insights. We here report our studies of a 22 year old male with features of PWS who has a *de novo *balanced reciprocal translocation t(4;15)(q27;q11.2). This is the first such case where the translocation breakpoints have been identified at the DNA sequence level. The cytogenetic breakpoint designations in this individual are identical to those in another male PWS-like case with t(4;15)(q27;q11.2), previously reported by Kuslich and colleagues [27] and restudied by Gallagher and colleagues [28], which raised the intriguing possibility of a recurrent translocation that may be facilitated by genomic repeats or other distinct molecular features.

In the present case, however, we mapped the breakpoint to *SNRPN *intron 17 (position on chr. 15: 22803227, UCSC Genome Browser, May 2004) that differs from that in the previous case (*SNRPN *intron 2). Furthermore, the breakpoint in our case is novel as it does not fall into one of the two previously described "breakpoint clusters" in intron 2 and exon 20a/intron 20 (Table 3 and Table 4). On chromosome 4 (chr. 4 123965881), the LTR retrotransposon LTR1B is spanning the breakpoint, and a short interspersed element (SINE), AluY, and a LINE element, L1M4, surround the breakpoint on chromosome 15 (Fig. 6). Interestingly, 39 bp upstream of the breakpoint on chromosome 15 starts a common 26-bp core sequence of Alu elements (Alu-DEIN) that has been shown to be involved in gene rearrangements and has homology with prokaryotic χ, an 8-bp sequence motif known to stimulate *recBC *mediated recombination in E. *coli*[37]. The core sequence is identical to sequences in the left arm of the consensus Alu element [38]. Sequence analyses of regions directly adjacent to translocation breakpoints has shown presence of the 26-bp Alu core sequence at or close (within 20–50 bp downstream or upstream) to the sites of recombination [36]. Therefore, this sequence might stimulate homologous and non-homologous recombination within the core or at nearby sites and could be the mechanism of recombination in the t(4;15) case reported here.

The translocation is *de novo*, as is true for all the previously described cases with translocation breakpoints involving the *SNPRN *gene. Given the PWS-like phenotype, the translocation was assumed to be of paternal origin. This assumption was confirmed by the expression studies. Paternal origin of the translocation was formally proven in 2 of the 5 previously reported cases [26,27].

### Karyotype – phenotype correlations

Two individuals with *SNRPN *intron 2 breakpoints were described as having classical PWS, meeting the major clinical criteria by age 3.5 years and additional minor clinical criteria [25,27]. The individuals with a breakpoint in *SNRPN *Exon 20/Intron 20 were described as having a milder or atypical form of PWS (Table 3 and Table 4). The weight gain started later than in classical PWS, at 7 and 5 years, respectively, for the patients described by Schulze et al. 1996 and Wirth et al. 2001, and at 8 years in our case. The characteristic facial features were absent in the case of Wirth et al. 2001, and also in the present case. But this is not a consistent feature in classical PWS, as in a retrospective evaluation of 90 molecularly-proven PWS cases, only 49% had the characteristic facial gestalt [39].

It is apparent from the review of the previously reported cases and the individual reported here (Table 3 and Table 4) that some of these translocation cases tend to have a milder, 'atypical' clinical picture, in comparison with classical PWS. There is not a complete absence of any of the major phenotypic features (neonatal hypotonia and feeding difficulty, hyperphagia from early childhood, obesity, cognitive compromise, hypogenitalism), but the degree of affection may be lower. None of the reported translocation cases had any additional features that might possibly be attributed to disruption of a gene on the reciprocal chromosome, and in no prior case had an attempt been made to identify a gene at this location.

Our sequence data mapped the breakpoint on chromosome 4 within intron 10 of a spliced polyadenylated transcript (BC045668). This unique cDNA clone represents a 3764 bp mRNA from a human testis library that does not appear to encode a protein. Its 5' end overlaps the interleukin 21 (IL21) transcript by 511 bp in the opposite direction (UCSC Genome Browser, May 2004). It appears unlikely that heterozygous disruption of this gene contributes to the phenotype in our patient.

### Translocation has no effect on imprinting center methylation and upstream genes

To assess whether the translocation event had affected the allele-specific methylation pattern at the imprinting center (IC), and/or to exclude a coincident imprinting defect, we carried out methylation studies of the *SNRPN *exon 1 region that revealed a normal bi-parental methylation pattern. Similar results were reported for each of the other five PWS individuals who had translocation breakpoints within the *SNRPN *gene. These results predict that expression of the genes located centromeric to the *SNRPN *exon 1/ IC region, *NDN*, *MAGEL2 *and *MKRN3*, should not be affected in these individuals. By studying t(4;15) fibroblasts by RT-PCR, we indeed found expression of all three genes. Previously, only *MKRN3 *was reported to be expressed in the three PWS translocation cases in which it was studied [20,25,27].

In t(4;15) lymphoblasts, the *SNRPN *transcript was detectable by RT-PCR and quantitative RT-PCR and found to extend all the way to exon 17. The major transcript that encodes the SNURF/SNRPN proteins terminates in exon 10 [20] and, therefore, should be unaffected by this t(4;15) translocation. With the caveat that studies on peripheral tissues, fibroblasts and lymphoblasts, may not accurately reflect gene expression in the brain, our results indicate that *SNURF/SNRPN *and the centromeric genes *MKRN3, NDN *and *MAGEL2 *are unlikely to play a prime role in the causation of PWS-associated features, although it remains an open question whether their loss or non-functioning might contribute to the more marked phenotypic expression that is seen in typical PWS.

### Genes downstream of the breakpoint are not expressed

With respect to expression of downstream transcripts, the reported results on LCLs with breakpoints in exon 20/intron 20 were consistent, whereas for the two patients with breakpoints in intron 2, the reported results were conflicting for expression of downstream transcripts IPW and PAR-1. In a re-evaluation of the t(4;15) case reported by Kuslich and colleagues [26], no expression of these transcripts and of the PWCR1/HBII-85 snoRNA cluster was detected by real-time quantitative RT-PCR [28].

Therefore, we focused our analysis on the snoRNAs and two ESTs in intron 20. As for the intron-encoded C/D box snoRNAs, HBII-13 and HBII-437 were expressed, and HBII-438A/B and HBII-85/PWCR1 were not. HBII-52 snoRNAs were not studied, as they are not expressed in the available tissues and have previously been excluded from contributing to the PWS phenotype [40,41]. The two ESTs in the large intron 20 that are highly expressed in brain tissues [42] were found to be expressed in a normal control LCL, but not in the t(4;15) LCL. This result suggests that these ESTs do not have their own promoter but are dependent on transcription from the *SNRPN *promoter that is located on the other translocation derivative in these cells. Therefore, these ESTs most likely represent stable derivatives of large alternatively spliced non-coding SNRPN transcripts.

## Conclusion

(1) Expression of the ESTs and/or C/D box snoRNAs that are located downstream of the translocation breakpoint is not necessary for establishing and maintaining the paternal-specific pattern of gene expression pattern that is controlled by the imprinting center upstream of the translocation breakpoint.

(2) The C/D box snoRNAs HBII-438A and PWCR1/HBII-85 are the only stable transcripts in this region that are disrupted in this t(4;15) PWS individual. As PWCR1/HBII-85 sequences are highly conserved between human and mice, while no copy of HBII-438A has been found in mouse, we conclude that the basis of PWS pathogenesis resides, in whole or in part, in the absence of PWCR1/HBII-85 snoRNA. *SNURF/SNRPN *and the centromeric genes *MKRN3, NDN *and *MAGEL2 *are unlikely to play a major role in the causation of PWS-associated features. While the function of known C/D box snoRNAs is to guide 2'- O -ribose methylation of mainly ribosomal RNA, these novel imprinted snoRNAs have no known target. They might be involved in a posttranscriptional regulation process of a gene or genes that – if non-functional – gives rise to the PWS phenotype.

## Competing interests

The author(s) declare that they have no competing interests.

## Authors' contributions

BS carried out the molecular genetic studies (RT-PCR, methylation assay, Southern blot analysis, and breakpoint analysis) and drafted the manuscript. MA carried out quantitative RT-PCR assays. EN performed the FISH analysis with BAC clones. DIF carried out the initial cytogenetic analysis. MR revised the clinical data and re-examined the patient.

HRS supervised the cell culturing, cytogenetic and FISH studies. RJMG diagnosed the patient, collected the clinical data and obtained skin and blood samples. UF conceived the study design, and coordinated its progress, supervised the work of BS and MA and prepared the final manuscript.

## Pre-publication history

The pre-publication history for this paper can be accessed here:



## Figures and Tables

**Table 1:** Primers and conditions for PCR

**RT-PCR **				
**Gene/Exon **	**fwd (5'-3') **	**rev (5'-3'); complement strand **	**size **	**T ann. **
ZNF127	GGG TTG CGG TTT TGC TAT TA	TTT CTC GTG TGC TTC AAT GC	168 bp	59C
MAGEL2	CTG AAG CCT GGG ACT TTC TG	GGA CCT TGG CCA CAA ACT TA	225 bp	59C
Necdin	GAA GAA GCA CTC CAC CTT CG	CCA TGA TTT GCA TCT TGG TG	164 bp	59C
SNRPN Ex 1–3	ATG GAG CGG GCA AGG GAT CGC	GGT ACA ACT GAC ACT CTT GG	124 bp	53C
SNRPN Ex 14/16	CTG CAA ACA TAG GAG ATG ATA GTT CC	CTT ATG AAA GCA CTG AGA TGA AGC C	459 bp	53C
SNRPN Ex 16/17	GAA AGT GAC CTA AAG AGT GTC ATT G	CTT GCA GTT GGA CAG CCG ACT C	515 bp	53C
SNRPN Ex 17/18	AGA TAT CTT TAA AAT TGA GTC TTC TGT CCA	TGA AGA TGC AGC ACT TTT GAA GAA	218 bp	53C
SNRPN Ex 19/20a	CAT TGT GCT TAT TTA CTA TTT TTG TAG ACG	CTG CAG GTG GTG ACC ATG TG	150 bp	53C
AK094315	TCT TCT CTA CCC TCA TTC CCA GC	TCG CTA CAC CCC TTT GCT TAT G	222 bp	53C
AB061718	AGG AGG GGT TCA AAG ATG C	CTG GTA AAC AAA CTG GTA AAG GTG	204 bp	50C
HBII-85/PWCR1	CGA TGA TGA GTC CCC CAT AAA AAC	CAG TTC CGA TGA GAA CGA CG	79 bp	53C
HBII-13	GGA TTT GTG ATG AGC TGT GTT TAC	GGA CTT CAG AGT AAT CAC GTT G	67 bp	54C
HBII-438A/B	GGA TCG ATG ATG AGA ATA ATT ATT G	GGA CCT CAG ATT GAC ATC TG	67 bp	53C
GABRB3	TCA GGC GGC ATT GGC GAT ACC	ATA AAA ACT TGA CAG GCA GAG	352 bp	52C
GABRA5	AAT ATT GCC TTA ATG TTT CTA	GCC TAT TCT ATT TCT TCG TGT	425 bp	48C
GABRG3	GCG TAT TCA CAT AGA CAT CTT G	GAT TGG TCA CTA CTG GTC TGG	188 bp	52C
GAPDH	TGG GCT ACA CTG AGC ACC AG	GGG TGT CGC TGT TGA AGT CA	50 bp	53C
				
**Quantitative RT-PCR **				
**Gene/Exon **	**fwd (5'-3') **	**rev (5'-3');complement strand **	**size **	**T ann. **

SNURF Ex2	ACG AAC TAC AGA ACA GCA CGT ACC	CTG CGT TTG ACT TGG ACT TCC	50 bp	60C
SNURF Ex3	TTC TCA GCA GCA GCA AGT ACC T	TGC CTC AGT TCA GCC TGG A	50 bp	60C
HBII-437	ATC ATT ATT TCT TGA ATT GG	CCC TCA CGC TCC CTT TGC	50 bp	60C
SNRPN Ex 14/15	CTG CAA ACA TAG GAG ATG ATA GTT CC	CAA AGA CGA TAA AAT GTT CCT TCT TG	50 bp	60C
SNRPN Ex 19/20a	GGA ACC ACC ATT TGT CTA TGA TCC	CTG CAG GTG GTG ACC ATG TG	50 bp	60C
HBII-438	ATA ATT GTC TGA GGA TGC T	GAT TGA CAT CTG GAA TGA GTC	50 bp	60C
HBII-85/PWCR1	TCG ATG ATG AGT CCC CCA TAA	CAT TTT GTT CAG CTT TTC CAA GG	50 bp	60C
				
**PCR to generate Southern probes in intron 17 **				
**Gene/Exon **	**fwd (5'-3') **	**rev (5'-3');complement strand **	**size **	**T ann. **

SB 1	ACC ATC AGT GAA TGA CCT GTT GC	CCC AGC CTC TTT CCT ATG TCT TG	565 bp	53C
SB 3	TGG TAA ACT GAT GAG AGC ACA GCC	GCC TGG GAG ACA GAA TGA GAA AC	416 bp	53C

**Table 2:** SNRPN and snoRNA expression analysis with quantitative RT-PCR

Amplification product	PWS	Normal control	t-PWS (4;15)	PWS triplication	t-PWS intron 2	t-PWS (4;15)
	LCL	LCL	LCL	LCL	FB	FB
SNURF Ex 2	0.0001	0.53	0.54	2	0.002	0.56
SNURF Ex 3	0.0002	1.1	1.43	6.4	0.0004	0.56
HBII-437	0.00003	0.86	1.13	4.9	0.00008	0.1
SNRPN 14/15	0.0013	4.7	4.91	17.23	-	-
SNRPN 19/20a	0.005	1.34	0.003	4.7	0.007	0.007
HBII-438	0.03	1.5	0.02	6.2	0.07	0.07
PWCR1/HBII-85	0.03	3.7	0.02	16.8	0.04	0.06

Sample identification: PWS: PWS with an IC microdeletion (patient E in [5]); t(4;15) PWS: the PWS case reported here; PWS-triplication: intrachromosomal triplication of the PWS region [33]; t-PWS intron 2: previously reported PWS case with t(4;15)(q27;q11.2) and breakpoint in *SNRPN *intron 2 [26, 27]; LCL, lymphoblastoid cell line, FB, fibroblast strain. The numbers represent the ratio of target product to GAPDH control product.

**Table 3:** Clinical findings associated with paternally-derived *de novo *reciprocal translocations involving *SNRPN*

	Breakpoint in *SNRPN *Intron 2		Breakpoint in *SNRPN *Exon 20/ Intron 20			Breakpoint in *SNRPN *Intron 17
	
	Sun et al. 1996	Kuslich et al. 1999	Schulze et al. 1996	Conroy et al. 1997	Wirth et al. 2001	Present case
Karyotype designations	46, XY, t(15;19) (q12;q13.41)	46, XY, t(4;15) (q27;q11.2)	46, XY, t(9;15) (q21;q12–q13)	46, XY, t(2;15) (q37.2;q11.2)	46, X, t(X;15) (q28;q12)	46 XY, t(4;15)(q27;q11.2)

Age of examination	3.5 years	3 years 3 months	29 years	4.5 years	18 years	22 years

**Major criteria **(each scores one point) from [1] as revised in [2].						

1. Neonatal central hypotonia	Floppy and lethargic in the first 6 months with poor suck (1pt.)	Hypotonicity, poor sucking reflex during infancy (1pt.)	Neonatal hypotonia (weak cry, poor suck) (1 pt.)	Neonatal hypotonia, lethargy, poor suck (1 pt.)	-	Reduced tone with poor head control, poor suck (1 pt.)

2. Infantile feeding problems/ failure to thrive		Failure to thrive (1pt.)	Feeding problems in infancy, failure to thrive (1 pt.)	Special feeding techniques, but no failure to thrive	-	Feeding problems, but no failure to thrive (1pt.)

3. Rapid weight gain between 1–6 years	Obesity starting at 6 months, hyperphagia (1 pt.)	Eating behavior leading to increased weight gain at age 2 yr (1 pt.)	Periodic excessive weight gain from age 7 yr	Onset of obesity at 1.5–2 yr with excessive appetite and food foraging (1 pt.)	Obesity began at 4–5 yr with hyperphagia and food foraging (1 pt.)	Late onset obesity (at approx. 8 yr)

4. Characteristic facial features	Narrow bifrontal diameter, almond-shaped eyes, down-turned mouth (1pt.)	Narrow bifrontal diameter, almond-shaped eyes, upslanted palpebral fissures (1 pt.)	Narrow bifrontal diameter, narrow face, small mouth, poor facial mimic (1pt.)	Narrow bifrontal diameter, squared nasal tip, downturned mouth (1 pt.)	-	-

5. Hypogonadism: genital hypoplasia, pubertal deficiency	Undescended testes (1 pt.)	Undescended small testes, hypogonadism (1 pt.)	Hypoplastic genitalia, incomplete gonadal maturation with delayed pubertal signs after age 16 yr (1 pt.)	Scrotum normal, penile length at 10^th ^%ile	Primary amenorrhea, hypoplastic uterus (1 pt.)	Undescended small testes, hypogonadism, delayed pubertal signs (1 pt.)

6. Mental retardation, developmental delay	Developmental delay (1 pt.)	Developmental delay (1 pt.)	Mental retardation, developmental delay/ learning problems (1 pt.)	Developmental delay, special school setting (1 pt.)	Slight developmental delay, school for mentally retarded children (1 pt.)	Developmental delay, special school setting (1 pt.)

Score	5 points	6 points	5 points	4 points	3 points	4 points

Blank cell = no information

- = absent

**Table 4:** Clinical findings associated with paternally-derived *de novo *reciprocal translocations involving *SNRPN *(continued)

	Sun et al. 1996	Kuslich et al. 1999	Schulze et al. 1996	Conroy et al. 1997	Wirth et al. 2001	Present case
**Minor criteria **(1/2 point each)						

1. Decreased fetal movement and infantile lethargy	Decreased fetal activity (0.5 pt.)	Decreased fetal movements (0.5pt.)		-	-	Slightly reduced fetal movements (0.5pt.)

2. Typical behaviour problems	Behavior problems (0.5pt.)	Temper tantrums, violent outbursts, obsessive-compulsive (0.5 pt.)	Aggressive outbursts, rigid personality, perseveration (0.5pt.)	Behavior problems with temper tantrums and severe aggressiveness (0.5 pt.)	Temper tantrums, violent outbursts after food restrictions (0.5pt.)	Temper tantrums, abnormal social behavior (0.5pt.)

3. Sleep disturbance, sleep apnea		Sleep disturbance, sleep apnea (0.5pt.)	Sleep disturbance (0.5pt.)			Sleep disturbance, amphetamine treatment from age 9 ys. (0.5pt.)

4. Short stature for the family by age 15 years			Short stature at the age of 15 (0.5pt.)	50–75^th ^percentile (0.5pt.)	151 cm (3^rd^%tile) (0.5pt.)	Height 155.7 cm at 16 years < 3^rd ^%tile (0.5 pt.)

5. Hypopigmentation	-	-	Hypopigmentation (0.5 pt.)		-	-

6. Small hands and /or feet for height age	Hand length 25^th ^percentile, finger length 10^th^%ile (0.5pt.)	-	Normal hands, but small feet (< 10^th^%tile) (0.5 pt.)		Short 3rd finger bilaterally	Hands 20^th ^%ile, feet 5th %ile (0.5pt.)

7. Narrow hands with straight ulnar border		-				-

8. Eye abnormalities: esotropia, myopia		-	Esotropia (0.5 pt.)	Alternating esotropia in infancy (0.5 pt.)	Left esotropia (0.5 pt.)	Esotropia (0.5pt.)

9. Thick viscous saliva		Viscous saliva (0.5pt.)		Thick viscous saliva (0.5 pt.)		-

10. Speech articulation defect		Articulation difficulty (0.5 pt.)		Poor articulation (0.5pt.)		-

11. Skin picking		Skin picking (0.5pt.)	Skin picking (0.5pt.)			Skin picking (0.5pt.)

Score (minor only)	1.5 points	3 points	3.5 points	2.5 points	1.5 points	3.5 points

Total Score	6.5 points	9 points	8.5 points	8.5 points	4.5 points	7.5 points

Blank cell = no information

- = absent
